# Diatoms of North America: Nomenclatural transfers within the Bacillariophyceae 1

**Published:** 2024-02-20

**Authors:** Mark B. Edlund, Sarah A. Spaulding, Ian W. Bishop, Marina Potapova, Sylvia S. Lee, Paula C. Furey, Elena Jovanovska

**Affiliations:** 1 St Croix Watershed Research Station, Science Museum of Minnesota, Marine on St Croix, MN 55047, USA.; 2 U.S. Geological Survey, INSTAAR, 4001 Discovery Drive, N202, Boulder CO 80303, USA; 3 U.S. Geological Survey, Southwest Biological Science Center, Grand Canyon Monitoring and Research Center, Flagstaff AZ, 86001 USA; 4 Academy of Natural Sciences, Drexel University, Philadelphia, PA 19103, USA; 5 Office of Research and Development, U. S. Environmental Protection Agency, Washington, DC, 20460, USA; 6 Department of Biology, St. Catherine University, 2004 Randolph Ave, St. Paul, MN 55105, USA; 7 Department of Palaeoanthropology, Senckenberg Research Institute and Natural History Museum, Senckenberganlage 25, 60325 Frankfurt am Main, Germany

The online diatom identification and ecological assessment guide, *Diatoms of North America* (https://diatoms.org), provides taxonomic, nomenclatural, and ecological information on over 1000 diatom species to date (Spaulding & al. 2021). In creating and maintaining species pages for the website and in supporting efforts to achieve taxonomic certification by the *Society for Freshwater Science* (https://freshwater-science.org/about/taxonomic-certification), issues have arisen regarding the identification, nomenclature, and systematic position (e.g. genus) of taxa. Here we propose several nomenclatural transfers necessitated by recent changes in the classification and phylogeny of diatoms (Ruck & al. 2016, Jahn & al. 2019, Wynne 2019).

***Surirella internationalis*** (Bahls) Spaulding, *comb. nov*.

Basionym: *Cymatopleura internationalis* Bahls, *Phytotaxa*, 82(1): 25, fig. 104. 2013 (‘*internationale*’).

PhycoBank Registration: http://phycobank.org/104259

Notes: Recent revisions based on molecular phylogenies of the keeled and canal raphe-bearing diatom genera *Surirella*, *Cymatopleura*, *Rhopalodia*, and *Iconella*, revealed monophyly of the genus *Surirella*. This necessitated nomenclatural transfers of many nested taxa within *Surirella* (Ruck & al. 2016), including the freshwater forms of *Cymatopleura*. However, *Cymatopleura internationalis* from Glacier National Park, Montana, USA, described by Bahls (2013), was not included in the transfers to *Surirella* proposed by Ruck & al. (2016) and Jahn & al. (2017).

***Gomphonella fogedii*** Edlund, *stat. et nom. nov*.

Replaced synonym: *Gomphonema olivaceoides* var*. densistriatum* Foged, *Natura Jutlandica* 10: 40, pl. VI [6]: fig. 5, 1963 (‘*densestriata*’).

PhycoBank Registration: http://phycobank.org/104260

Notes: Recent revisions based on molecular and morphological phylogenies of *Gomphonema* and its allies (Jahn & al. 2019) resulted in the recognition of a monophyletic grouping around *Gomphonema olivaceum* (Hornemann) Ehrenberg and allies. *Gomphonema olivaceum* was originally described as *Ulva olivacea* Hornemann (1810: fasc. 24; pl. MCCCCXXIX [1429]), which was transferred as *Gomphonella olivacea* (Hornemann) Rabenhorst (1853: 61, pl. IX [9]), the generitype of *Gomphonella* Rabenhorst, 1853. As a result, Jahn & al. (2019) resurrected the genus *Gomphonella* to define this monophyletic grouping. One taxon that was not transferred on the basis of this resurrected name was Foged’s *Gomphonema olivaceoides* var*. densistriatum*, which has all the characters (Foged 1963, Bishop 2017) of the resurrected genus *Gomphonella*, necessitating this transfer and a new name at the species level as *Gomphonella densistriata* (Levkov) R.Jahn & N.Abarca is already occupied.

***Delicatophycus tundraphilus*** (Bahls) Edlund, *comb. nov*.

Basionym: *Cymbopleura tundraphila* Bahls, *Diatoms from western North America 1. Some new and notable biraphid species*. p. 16, fig. 70, 2017.

PhycoBank Registration: http://phycobank.org/104261

Notes: Recent recognition that several generic diatom names were based on descriptive or adjectival terms led to new validated names, including *Decussatophycus* (Guiry & Gandhi. 2019), *Delicatophycus* (Wynne 2019), *Pulchellophycus* (Edlund & Wynne 2019), and others (e.g. Blanco & Wetzel 2016, Turland & al. 2018). However, *Cymbopleura tundraphila*, described by Bahls (2017) from Nunavut, Canada (Coppermine River, September Mountains Lake), shares diagnostic features (Bahls 2018) with the recently validated genus *Delicatophycus*, justifying this transfer, as suggested by Bahls (2019: 22).

The authors represent the Editorial Review Board of the *Diatoms of North America* web resource (diatoms.org). The web resource is supported by the US Environmental Protection Agency, the USGS, the National Park Service Great Lakes Network, and the Institute of Arctic and Alpine Research at University of Colorado Boulder. This project was funded in part by the National Park Service grant P20AC00364 to MBE. The views expressed in this publication are those of the authors and do not necessarily reflect the views or policies of the US Environmental Protection Agency. Mention of trade names or commercial products does not constitute endorsement or recommendation for use by the U.S. Government.
